# Microsporidia persistence in host impairs epithelial barriers and increases chances of inflammatory bowel disease

**DOI:** 10.1128/spectrum.03610-23

**Published:** 2023-12-27

**Authors:** Jiangyan Jin, Yunlin Tang, Lu Cao, Xue Wang, Yebo Chen, Guozhen An, Huarui Zhang, Guoqing Pan, Jialing Bao, Zeyang Zhou

**Affiliations:** 1 The State Key Laboratory of Resource Insects, Southwest University, Chongqing, China; 2 Chongqing Key Laboratory of Microsporidia Infection and Control, Southwest University, Chongqing, China; University of Edinburgh, Midlothian, United Kingdom

**Keywords:** microsporidia, epithelial barrier function, inflammatory bowel disease, gut microbiota, tight junctions

## Abstract

Microsporidia are intracellular fungus-like pathogens and the infection symptoms include recurrent diarrhea and systematic inflammations. The major infection route of microsporidia is the digestive tract. Since microsporidia are hard to fully eliminate, the interactions and persistence of the pathogen within epithelium may modulate host susceptibility to digestive disorders. In this study, both *in vitro* and *in vivo* infection models were applied. The alterations of epithelial barrier integrity, permeability, and tight junction proteins after microsporidia infection were assessed on MDCK/Caco-2 monolayers. The fecal intestinal microbiota and tissue alterations after microsporidia infection were assessed on C57BL/6 mice. Moreover, the susceptibility to develop dextran sulfate sodium (DSS)-induced inflammatory bowel diseases (IBDs) was also analyzed by the murine infection model. The results demonstrated that microsporidia infection increased epithelium permeability, weakened wound healing capability, and destructed tight junction protein zonula occludens-1. Microsporidia infection also dysregulates intestinal microbiota. These impairing effects of microsporidia increased host vulnerability to develop enteritis as shown by the murine model of DSS-induced IBD. Our study is the first to elucidate molecular mechanisms of the damaging effects of microsporidia on host epithelium and pointed out the cryptic threats of latent microsporidia infection to public health as reflected by the increased chances of developing more severe diseases.

**IMPORTANCE:**

Microsporidia are widely present in nature and usually cause latent and persistent infections in hosts. Given the fact that the digestive tract is the major infection route, it is of great importance to explore the consequences of microsporidia infection on the intestinal epithelial barrier and the risks to the host. In this study, we demonstrated the destructing effects of microsporidium infection on epithelial barriers manifested as increased epithelial permeability, weakened wound healing ability, and disrupted tight junctions. Moreover, microsporidia made the host more susceptible to dextran sulfate sodium-induced inflammatory bowel disease. These findings provide new evidence for us to better understand and develop novel strategies for microsporidia prevention and disease control.

## INTRODUCTION

Microsporidia are obligate intracellular eukaryotic pathogens. They cause microsporidiosis in humans and other vertebrates with symptoms such as recurrent diarrhea, but more frequently the infections are asymptotic and the persistence within hosts is difficult to completely eliminate (1–3). The host range of microsporidia is extremely large, from vertebrates to invertebrates (4). By far, there are 17 microsporidia species that can infect humans and the most commonly diagnosed and studied are the four species, *Encephalitozoon hellem* (*E. hellem*), *Encephalitozoon cuniculi* (*E. cuniculi*) and *Encephalitozoon intestinalis* (*E. intestinalis*), and *Enterocytozoon bieneusi* (*E. bieneusi*) (5, 6). The major infection route of microsporidia is through ingestion by water or food, therefore the digestive tract is the front line of host-pathogen interactions (7, 8).

The intestinal epithelial barrier, consisting of monolayers of intestinal epithelial cells and adjacent cells/tissues can effectively prevent the passage of macromolecular substances (9, 10). In addition, epithelial barrier together with surrounding immune cells also regulates the immune response against invading pathogens (11). Adjacent epithelial cells are connected through tight junctions, of which zonula occludens-1 (ZO-1) is an essential component protein (12, 13). MDCK and Caco-2 cells are common cell models to study barrier integrity, because of the ability to form epithelium monolayers and the abundant contents of tight junction proteins (14–16). ZO-1 belongs to the membrane-associated guanylate kinase homologs family, which are scaffold proteins involved in numerous protein-protein interactions through specific conserved domains (17). The abnormal expressions or localizations of ZO-1 were usually associated with abnormal epithelial cell functions, immune cell recruitments, cytokine expressions, inflammatory bowel disorders, or even tumor progressions (18–20). In addition to reside and proliferate within the host digestive tract, microsporidia would often penetrate the epithelium barrier, disseminate throughout the host, and cause systematic infections (21–23).

Inflammatory bowel disease (IBD) is the collective name for Crohn’s disease and ulcerative colitis, and the symptoms include diarrhea, fever, blood in stool, and sometimes fatal to hosts (24). The exact cause of inflammatory bowel disease remains unknown; however, researchers have identified the epithelial barrier functions, the gut microbiota, and chronic local inflammation were essential factors for the disease progression (25, 26). Inflammatory bowel disease is more common after gastrointestinal infections (27, 28). Considering the facts that altered microbiota may be a causative agent in inflammatory bowel disease and the persistence of microsporidia in the epithelium, it is of great importance to investigate the impairing effects of microsporidia on epithelial barriers and whether the persistence promotes IBD development in the host.

Here, in this study, we utilized both *in vitro* and *in vivo* infection models to elucidate the impairing effects of microsporidia on intestinal epithelium barriers and investigate the pathogenesis of IBD after microsporidia infection. Our study indicated that the persistence of microsporidia may present as a great threat to public health and our findings may help to develop novel strategies of disease prevention and control.

## RESULTS

### Microsporidia infection impaired epithelial barrier integrity

**FIG 1:**
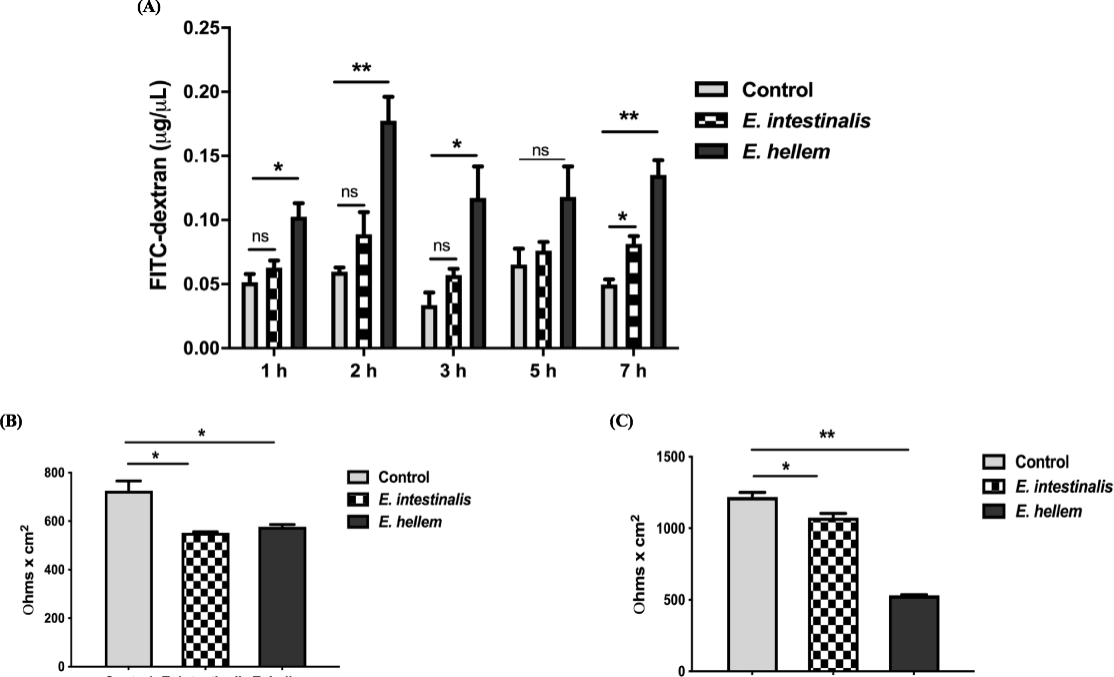
Microsporidia infection impaired host epithelium barrier integrity. (**A**) The transwell permeabilities of MDCK monolayer against FITC-dextran were significantly increased after microsporidia infection, specifically *E. hellem* (one hpi, two hpi, three hpi, seven hpi) and *E. intestinalis* (seven hpi). (**B**) Trans-epithelial electrical resistances of MDCK monolayers were significantly decreased after microsporidia, *E. hellem* and *E. intestinalis*, infections. (**C**) Trans-epithelial electrical resistances of Caco-2 monolayers were significantly decreased after microsporidia, *E. hellem* and *E. intestinalis*, infections (*n* = 9/group, *P < .05, **P < .01). FITC, fluorescein isothiocyanate.

The major infectious route for microsporidia is the digestive tract; therefore, we first investigated whether the infection impairs host epithelial barrier integrity. Here, we used two representative microsporidia species of *Encephalitozoon* spp., *E. hellem* as well as *E. intestinalis*, which are both commonly diagnosed zoonotic microsporidia species. MDCK cells were cultured as monolayers to mimic the host epithelium then infected by *E. hellem* or *E. intestinalis*, respectively, and incubated for 2 days to let the pathogens reside and proliferate within the host cells. Fluorescein isothiocyanate (FITC)-dextran was then applied to the upper chamber of the cell culture, and the fluorescence in the bottom chamber was measured to reflect the epithelium permeability. Results showed that the permeability of monolayers was significantly increased after microsporidia infection (Fig. 1A).

The integrity of epithelium was reflected by not only permeability but also the trans-epithelium electrical resistance (TEER). To verify, the MDCK and Caco-2 cells were cultured as monolayers, *E. hellem* and *E. intestinalis* were applied for infections, respectively, as described above. The TEERs were measured and compared, and results demonstrated that TEERs of either MDCK or Caco-2 were significantly decreased after microsporidia infection (Fig. 1B and C). Taken together, the increased trans-well permeability to fluorescent dye and the decreased trans-epithelium electrical resistance all indicated that microsporidia could severely impair host epithelium barrier integrity.

### Microsporidia infection destructed inter-cellular tight junctions

**FIG 2:**
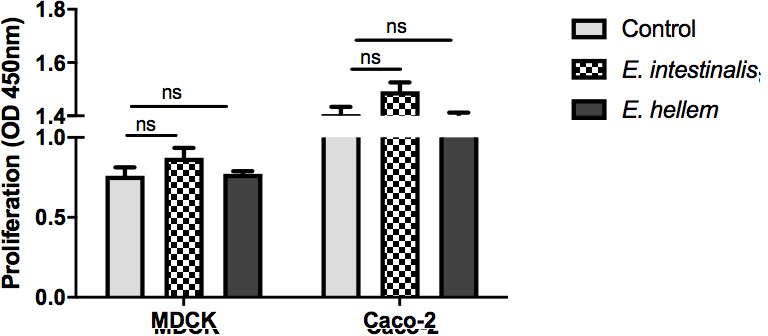
Microsporidia infections have no inhibitory effect on cell proliferation. The cell proliferation rates of MDCK and Caco-2 cells, with or without microsporidia infections, were measured by CCK8 assay (ns = no significance). The optical density (OD) at 450 nm is commonly used in cell proliferation assays (e.g. CCK8 assay). Normal range usually is at 0-3.

**FIG 3:**
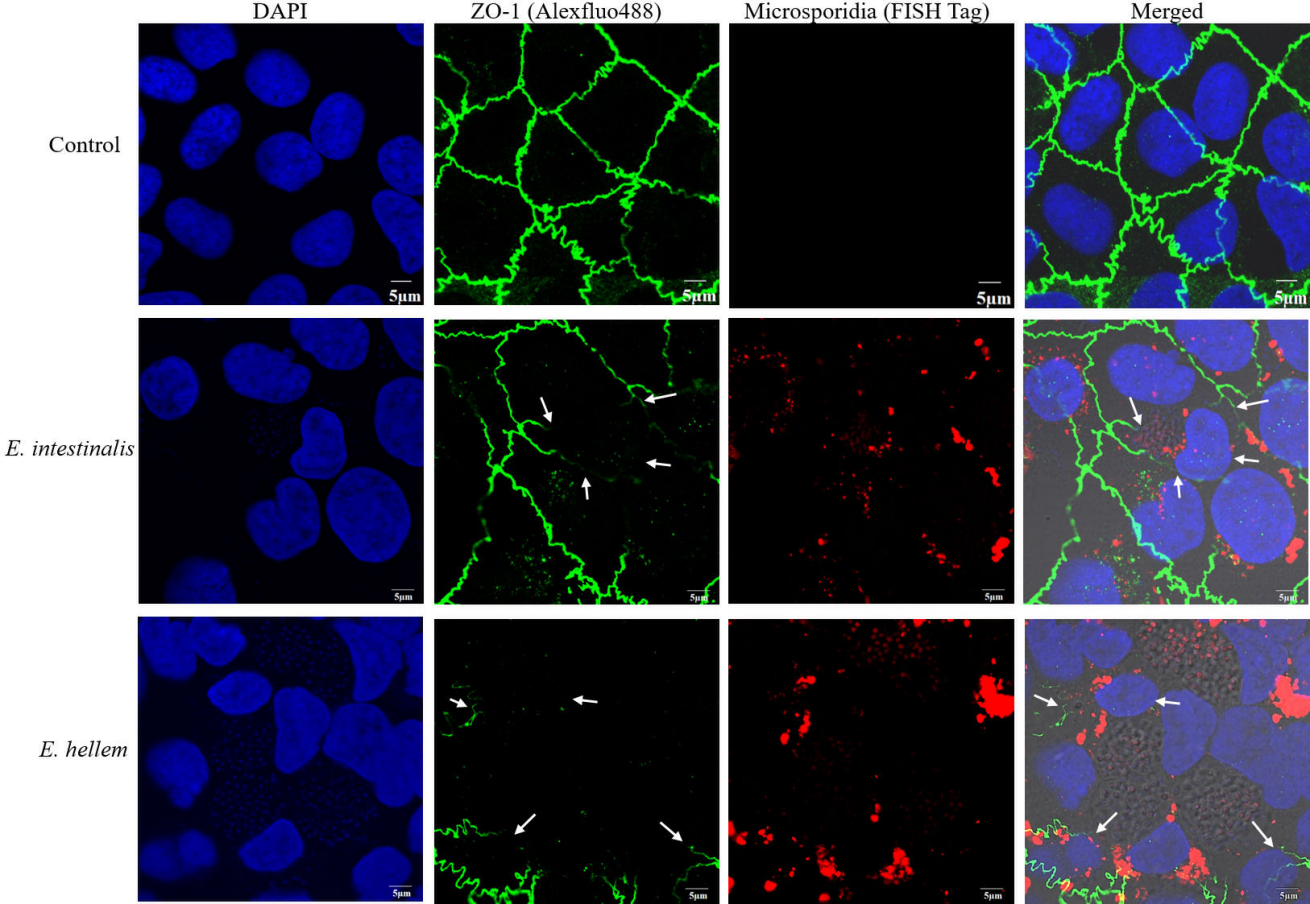
Microsporidia disrupted the structure of inter-cellular tight junctions of the epithelium monolayer. Epithelial cells Caco-2 cells were cultured to monolayer and then infected by microsporidia for 48 h, *E. hellem* and *E. intestinalis*, respectively. The inter-cellular structures were assessed by immunofluorescent staining. The successful infections/persistence of microsporidia were confirmed by microsporidia-specific rRNA FISH probes staining (red color) and forming spores in the merged images. The epithelial cells were not disturbed as shown by DAPI staining (nucleus, blue color). But, the inter-cellular tight junctions were severely disrupted, as shown by ZO-1 staining (green color) and arrows (scale bar = 5 µm). DAPI, 4′,6-diamidino-2-phenylindole; FISH, fluorescent in situ hybridization; ZO-1, zonula occludens-1.

Now, we have proved that microsporidia infection would significantly impair epithelium barrier functions as reflected by increased permeability and decreased TEER. To elucidate the molecular mechanism of the impairing effects, we first assessed the proliferation of cells by cell counting kit-8 (CCK8) assay. Results showed that microsporidia infection did not cause cell death nor delayed cell proliferation (Fig. 2). Therefore, the impairing effects of microsporidia on host epithelial barriers would be due to molecular basis such as destruction of tight junctions. We used immunofluorescent assays to verify the integrity of tight junctions, especially the essential component protein ZO-1 in epithelial cells. It is exciting to find that the continuous structures and localizations of ZO-1 were severely damaged by microsporidia infection (Fig. 3).

### Microsporidia infection weakened epithelium wound healing

**FIG 4:**
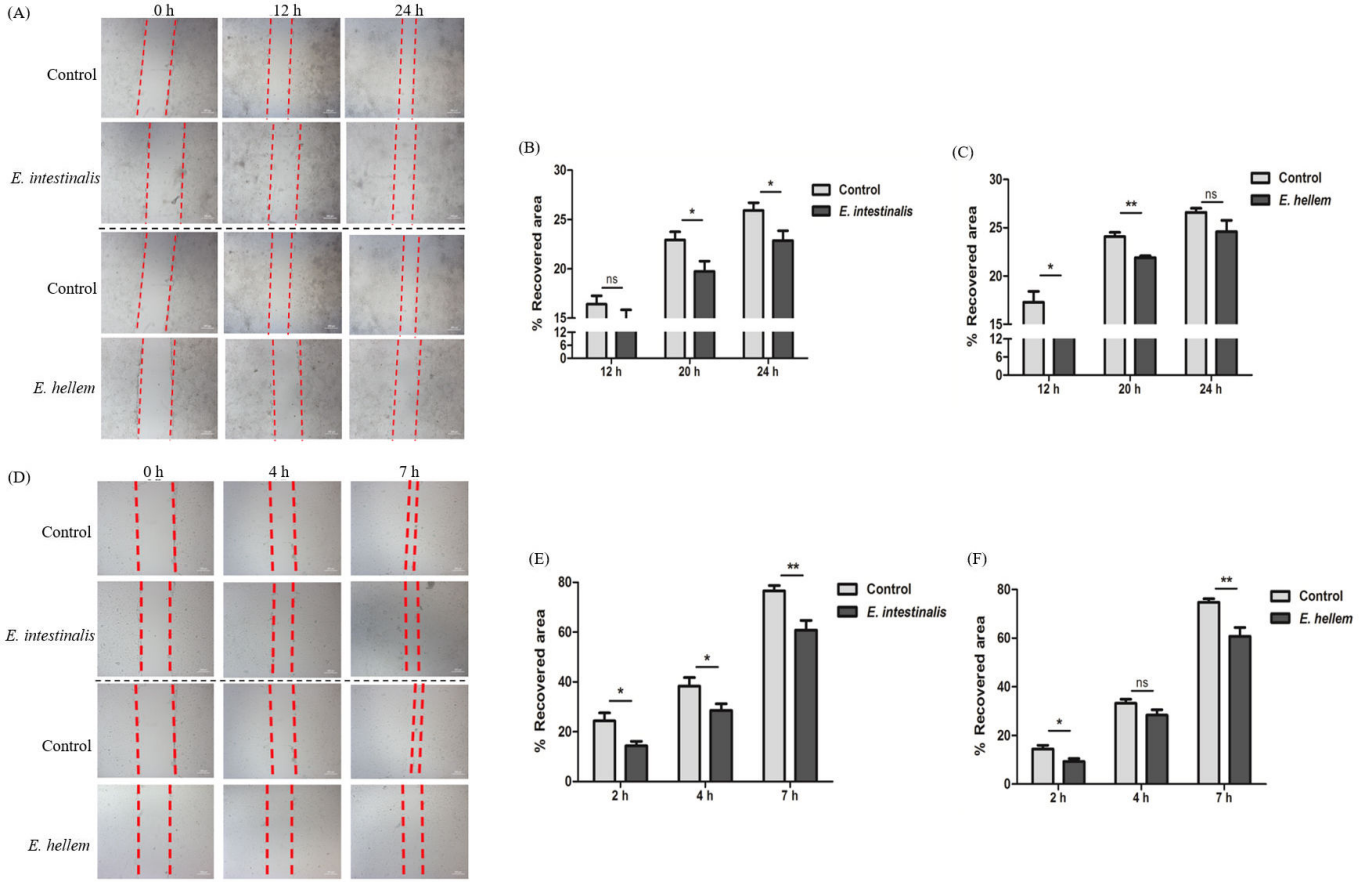
Microsporidia weakened epithelium wound healing. Epithelial cells MDCK and Caco-2 cells were cultured to monolayer and then infected by microsporidia *E. hellem* and *E. intestinalis*, respectively, for 48 h. The monolayers were scratched by the pipette tip, and the wound healing process was recorded and calculated. (**A**) Wound healing process of Caco-2 monolayers after *E. intestinalis* or *E. hellem* infection was significantly weakened. Red dashes lined the edges of the wounds at representatively 0, 12, and 24 h. (**B) and (C**) Quantifications of recovery rates at 12, 20, and 24 h after scratch. (**D**) Wound healing process of MDCK monolayers after *E. intestinalis* or *E. hellem* infection was significantly weakened. Red dashes lined the edges of the wounds at representatively 0, 4, and 7 h. (**E) and (F**) Quantifications of recovery rates at 2, 4, and 7 h after scratch (scale bar = 200 µm) (ns = no significance, *P < .05, **P < .01, *n* = 15/group).

Since microsporidia infection significantly impaired epithelial barrier integrity and destruct tight junctions, it is reasonable to infer that the epithelium wound healing ability would also be impaired. To verify, we cultured Caco-2 and MDCK cells as monolayers, scratches were introduced, and the healing processes were monitored and compared between microsporidia infected and un-infected groups. As shown by images and quantification results, microsporidia infections significantly delayed the epithelium wound healing processes (Fig. 4).

### Microsporidia infection disturbed host gut microbiota

**FIG 5:**
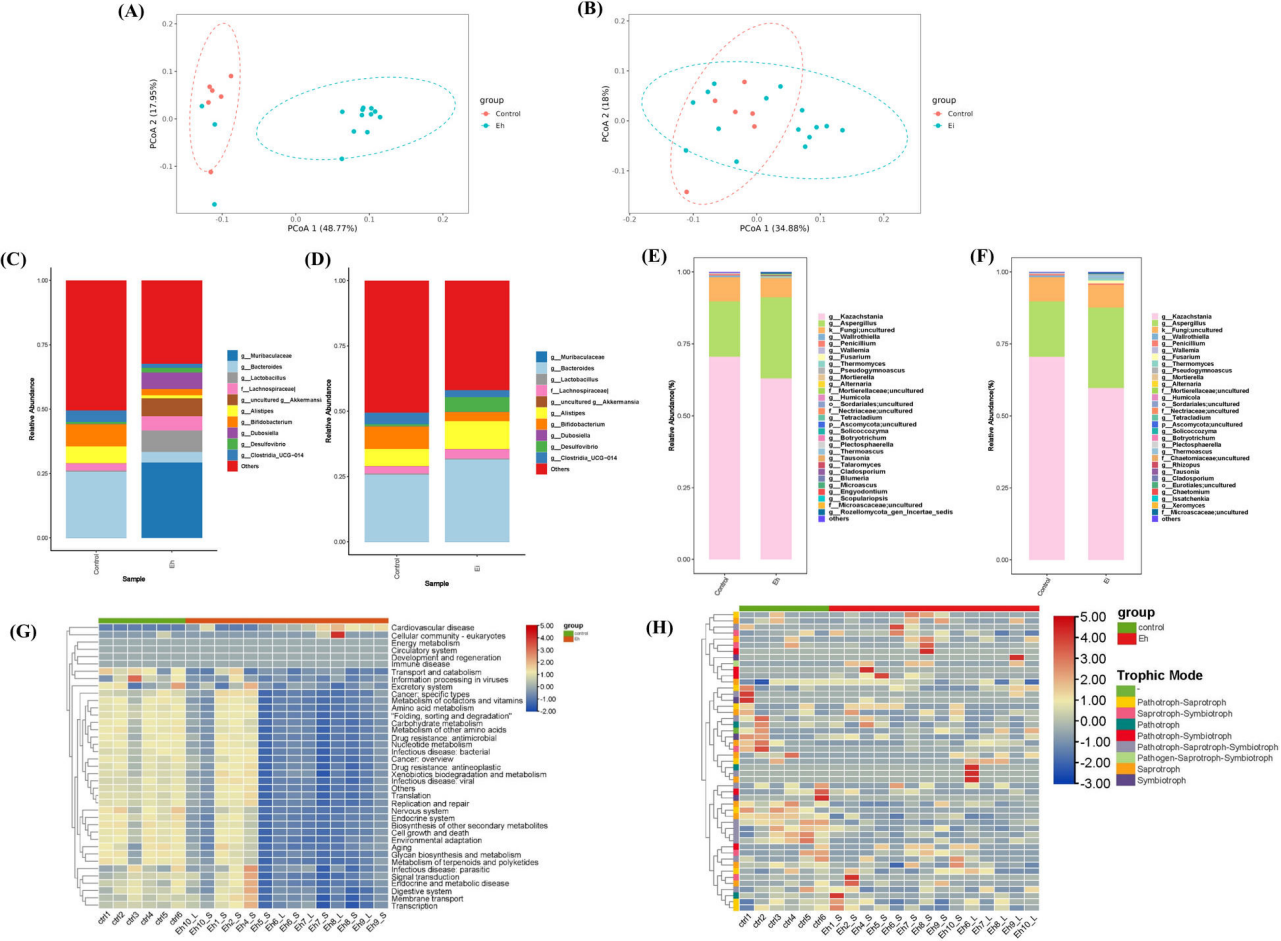
Microsporidia disturbed host gut microbiota. (**A**) Beta diversity analysis. PCoA score plot based on Weighted UniFrac metrics. Each point represents the mean principal component scores of each sample in a group. Microsporidia infections lead to significant discrete groups between *E. hellem*-infected and un-infected controls. (**B**) Beta diversity analysis. Microsporidia infections lead to discrete groups between *E. intestinalis*-infected and un-infected controls. (**C**) The composition of the gut bacteria changes after *E. hellem* at the genus level. As shown, relative abundances of *Bacteroides* spp. (26% vs 4%, *P* < 0.001), *Bifidobacterium* spp. (9% vs 2%, *P* < 0.01), and Clostridia_UCG-014 (4% vs 2%, *P* < 0.01) were decreased, while *Muribaculaceae* spp. (0% vs 29%, *P* < 0.01), *Lactobacillus* spp. (1% vs 8%, *P* < 0.05), and *Akkermansia* spp. (0% vs 7%, *P* < 0.01) were enriched. (**D**) The composition of the gut bacteria changes after *E. intestinalis* at the genus level. As shown, relative abundances of *Bifidobacterium* spp. (9% vs 4%, *P* < 0.01) were reduced. (**E**) The composition of the gut fungus changes after *E. hellem* at the genus level. As shown, relative abundances of *Kazachstania* spp. (71% vs 63%) decreased, while some other such as *Aspergillus* spp. (19% vs 28%) were enriched. (**F**) The composition of the gut fungus changes after *E. intestinalis* at the genus level. As shown, relative abundances of *Kazachstania* spp. (71% vs 60%) decreased, while *Aspergillus* spp. (19% vs 28%) were enriched. (**G**) Heat map of gut bacterium genes functions after microsporidia infection, as represented by *E. hellem* infection. Patterns showed that infection lead to down-regulation (blue color) of functions associated with metabolism, cellular response, and adaptations. (**H**) Heat map of gut fungus genes functions after microsporidia infection, as represented by *E. hellem* infection. Patterns showed that infection lead to down-regulation (blue color) of functions associated with saprothroph, pathotroph-symbiotroph, and pathotroph-saprothroph.

Microsporidia infection is usually hard to fully eliminate, the spores persist within host digestive tract. It is of great importance to investigate the effects of microsporidia on host gut microbiota. Beta diversity analysis showed that both *E. hellem* and *E. intestinalis*, especially the *E. hellem* infections, caused microbiota cluster separation from controls, indicating that microsporidia infection disturbed the structure of the intestinal microbiota of mice (Fig. 5A and B).

To further understand the alteration effects, the relative abundances of both bacterial and fungal microbes were analyzed. Results showed that gut bacteria such as *Bifidobacterium* spp., *Bacteroides* spp., and *Clostridia*_UCG−014 were significantly reduced (*P* < 0.01) by microsporidia infection, while some other bacteria, including *Muribaculaceae* spp., *Lactobacillus* spp., and *Akkermansia* spp. were enriched (Fig. 5C and D). For the fungal microbiota, microsporidium infection significantly enriched genus such as the *Aspergillus* spp., some species are known for the pathogenesis of IBD, while decreased some genus such as the *Kazachstania* spp., which are associated with ameliorates atopic reactions (Fig. 5E and F). Furthermore, the KEGG database and PICRUSt2 tool were utilized to analyze the gene functions and alterations of gut microbiota, and the graphic results demonstrated that microsporidia infection dysregulated various functions of microbiota. For instance, as shown in Fig. 5G and H , *E. hellem* infection down-regulated the bacterium genes that are associated with excretory, metabolism, antimicrobial resistance, environmental adaption, and so on; and also dysregulated majorly down-regulated some the fungus genes associated with nutrient cycling, symbiosis of residential microbes and pathogen-host interactions, represented in figures as saprotroph, pathotroph-saprotroph, and pathotroph-symbiotroph.

Taken together, our data demonstrated that microsporidium infection significantly disturbed the structures of the host’s gut microbiota thus possibly promoting the development of certain intestinal tract inflammations or disorders.

### Microsporidia persistence exacerbates IBD progression

**FIG 6:**
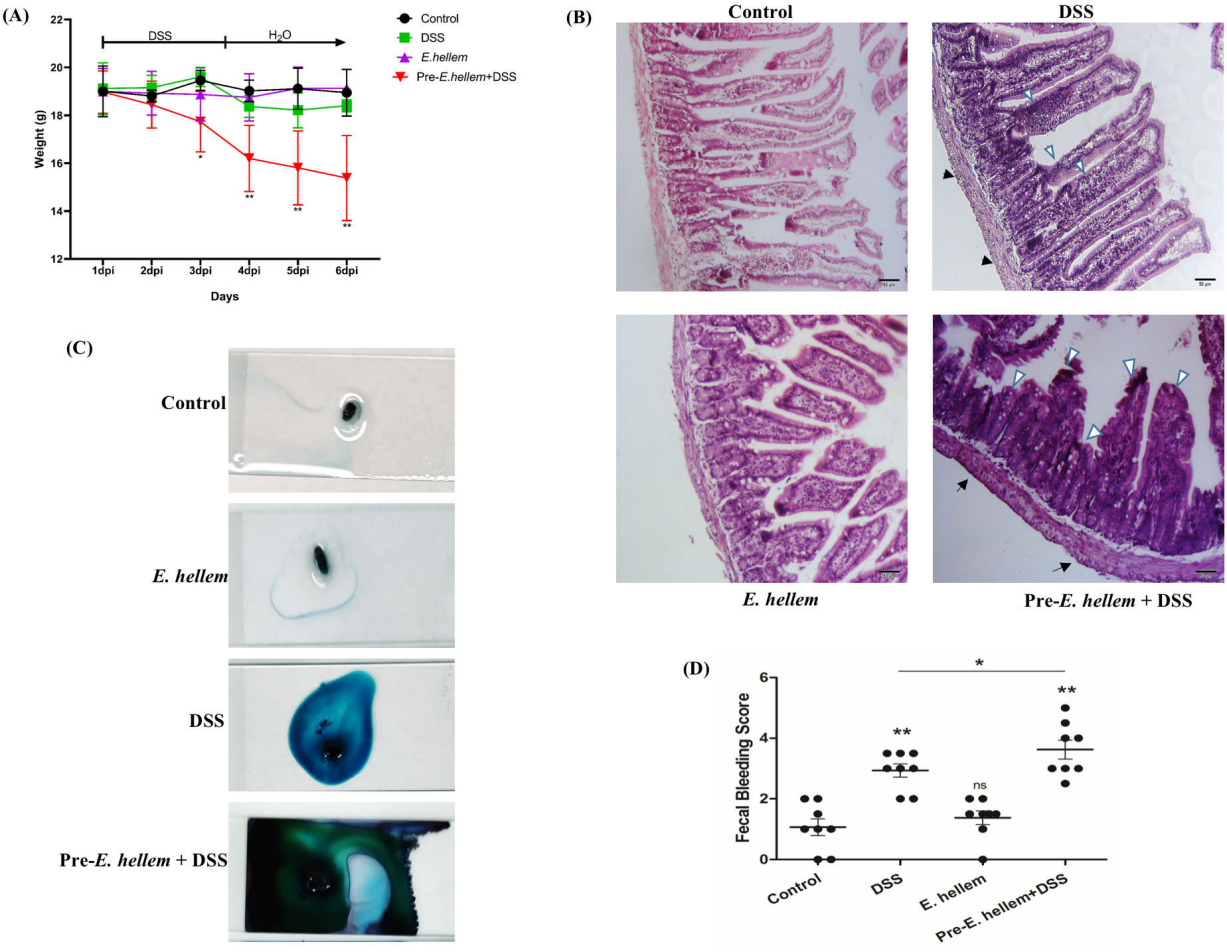
Microsporidia exacerbated IBD progression. The mouse enteritis model was conferred by dextran sulfate sodium (DSS, 3.5%). (**A**) Body weights of uninfected, *E. hellem* infected, DSS treated and *E. hellem* pre-infection+DSS treated groups of mice were measured. Pre-*E*. *hellem*+DSS treatment caused the most weight loss compared to other groups. (**B**) Histopathological analysis of mice intestines. The tissue sections were subjected to H&E stains. Compared to DSS treatment, pre-*E*. *hellem*+DSS treatment caused more edema of submucous layer (arrows), more infiltration of inflammatory cells, and distortions of intestinal epithelial cells (arrow heads). (**C) and (D**) Fecal bleeding records and scores. Feces of all groups of mice were collected and bleeding was measured with the fecal occult blood detection kit. Scores were based on the color of detection results. *E. hellem* infection alone has no effects on fecal bleeding, while pre-*E*. *hellem*+DSS treatment caused the most severe bleeding (ns = no significance, *P < .05, **P < .01, *n* = 4 for control group, *n* = 5 for DSS-treatment group, *n* = 6 for *E. hellem*-infected and Pre-*E*. *hellem*+DSS group, respectively).

Since the pathogenesis of IBD is highly associated with altered microbiota, epithelium barrier damages, and chronic inflammation, we thus infer the persistence of microsporidia would increase the susceptibility of the host to IBD. Here, we used DSS to induce the disease in mice and results demonstrated that the microsporidia pre-infection in mice would significantly increase the host weight loss (Fig. 6A), intestinal tissue damage (Fig. 6B), and fecal bleedings (Fig. 6C and D), thereby exacerbated IBD process.

## DISCUSSIONS

The current study verified the destruction of intestinal epithelial integrity and function by microsporidia infection, as demonstrated by the increased epithelium permeability, the decreased trans-epithelial resistance, and the deformation of the tight junction proteins such as ZO-1. The consequent intestinal damage and microbiota disturbance lead to increased host vulnerability to enteritis, as manifested by DSS-induced inflammatory bowel diseases. We utilized *E. hellem* and *E. intestinalis*, two commonly diagnosed digestive tract-infecting microsporidia species with comparable endemic rates, in our study to get a comprehensive view of the effects of microsporidia on epithelium barriers (29, 30). Our findings may help to explain the recurrent diarrheal and other intestinal abnormalities after microsporidia infection on molecular basis. Our study also shed light on the potential threat of microsporidia to public health, as the prolonged persistence within the host would definitely increase the chances of developing other intestinal enteritis and may cause other disorders.

Microsporidia infection in hosts presents primarily with diarrhea symptoms (31–33). It is known that diarrheal is usually caused by disturbed epithelium barriers and inflammations of epithelium, lamina propria, and surrounding cells (34, 35). Considering the fact that the obligated intracellular pathogens microsporidia persist and proliferate within host epithelial cells, it is of great interest to elucidate molecular mechanisms of microsporidia manipulating epithelium functions and possible consequences. Some intracellular pathogens affect the host cell’s life cycle, proliferation, or cell death. In comparison, our study revealed that the persistence of the “mild” intracellular pathogen microsporidia within epithelial cells does not affect the proliferation of host cells (36–38). Instead, the inter-cellular connects maintained by tight junctions were severely disrupted by microsporidia. Studies have proved that the successful wound healing process is accomplished by a combination of events such as the proliferation of cells, the migration abilities as well as the wound contraction or remodeling, and sometimes wound healing is accomplished without affecting cell proliferation (39–41). That explained the results of the severely impaired epithelium barrier integrity and the wound healing capability after microsporidia infections and attributed to the development of intestinal disorders.

In addition to the disrupted barriers, the gut microbiota was also proved to be disturbed by microsporidia infection. The composition, relative abundance, and structures of gut microbiota are the key factors and may be the sculptors of the development of intestinal disorders (42, 43). In our study, we proved that the biodiversity of intestinal microbiota was significantly decreased after microsporidia infection. The composition and relative abundances of microbiota also changed. The genus associated with beneficial and relieving effects were decreased, such as the *Bifidobacterium* spp. and *Bacteroides* spp. For instance, *Bifidobacterium longum* regulates oxidative stress by enhancing the body’s antioxidant activity and regulating the production and accumulation of reactive oxygen species (ROS), thereby reducing the symptoms of IBD (44, 45). In addition, some fungal microbes were significantly enriched after microsporidia infection. For instance, *Aspergillus* spp. and some species such as *Aspergillus sydowii* are considered emerging pathogens and have proven to evoke IBD (46). Therefore, it is reasonable to infer that the infection of microsporidia increased the chances of initiation and development of IBD. Our model of DSS-induced IBD in *E. hellem* pre-infected mice confirmed the inference, that the persistence of microsporidia aggravates enteritis progressions.

Due to the fact that microsporidia can persist within the host for a long period of time and usually are asymptomatic, the potential threats to the host and the public have long been underestimated. Accumulating studies have now revealed the co-infections of microsporidia with other vicious pathogens such as HIV and *M. tuberculosis*. The outcomes were usually more severe compared to single pathogen infections and sometimes fatal (32, 47). The molecular mechanisms of exacerbation have not been fully elucidated yet. Our study may shed light on the molecular basis. Microsporidia damaged epithelium barriers, and disrupted microbiota species which are essential for host immune homeostasis. All these may lead to weakened host barrier defences such as infiltrating leukocytes and residential phagocytes which are essential for local and systemic immune responses (48, 49).

Taken together, we verified in this study that microsporidium infection damaged the intestinal epithelial barrier integrity and functions through multifaceted evaluations, and further found that microsporidium infection aggravated the occurrence of IBD through DSS-induced mouse inflammation model. In conclusion, our study was the first to elucidate the mechanism and long-term impact of microsporidium infection on the host digestive tract and potentially systemic inflammations.

## MATERIALS AND METHODS

### Pathogens

*Encephalitozoon hellem* (*E. hellem*) strain (*ATCC 50504*) and *Encephalitozoon intestinalis (E. intestinalis*) are gifts of Prof. Louis Weiss (Albert Einstein College of Medicine, USA). Spores were inoculated and propagated in rabbit kidney cells (RK13, *ATCC CCL-37*) and human fibroblast cells (HFF, *ATCC SCRC-1041*), cultured in minimum essential medium Eagle (MEM) with 10% fetal bovine serum (FBS) (ThermoFisher, USA). The spores were collected from culture media, purified by passing them through 5 µm size filter (Millipore, Billerica, MA), and stored in sterile distilled water at 4°C. Spores were counted with a hemocytometer before usage.

### Cell lines

Human colon epithelial cancer cells (Caco-2, *ATCC HTB-37*) and Madin-Darby canine kidney cells (MDCK, *ATCC CCL-34*) were cultured in Dulbecco’s modified Eagles medium (DMEM) (Gibco, USA) containing 10% FBS (Gibco, Australia) with 5% CO_2_ at 37°C.

### Animals

Wild-type C57BL/6 mice (6-week, female) were reared in an animal care facility according to Southwest University-approved animal protocol (SYXK-2017–0019). At the end of the experiment, all mice were euthanized using carbon dioxide narcosis and secondary cervical dislocation.

### Microsporidia infection

*In vivo* infection of microsporidia to wild-type mice was achieved by adopting the following method. Previously, mice were transiently pre-treated with dexamethasone (Aladin, Cas 2392–39-4, China) to increase infection rates (50–53). In the current study, the dexamethasone pre-treatment was not necessary. In addition, 1 × 10^7^ spores/mice/day were inoculated into mice (intra-peritoneal) for 2 days. At the endpoint of the experiment, mice were sacrificed by CO_2_ inhalation. Samples of blood, urine, faeces, and organs were collected for further investigations.

*In vitro* infection of microsporidia was achieved by adding spores (30:1/spores: cells) to MDCK or Caco-2 cell cultures.

### Permeability assay

MDCK cell monolayers were cultured on inserted filters and then infected by microsporidia. At 48 h post-infection, the apical medium was replaced by 100 µg/mL of FITC-dextran (10 kDa) at. The basal chamber medium was collected at 1, 2, 3, 5, and 7 h of incubation at 37℃. The fluorescence in the medium was measured by a spectrophotometer (Molecular Devices, USA) at λex495 nm and λem 520 nm (54).

### Trans-epithelial electrical resistance

Caco-2 and MDCK cells were seeded on filters until reaching confluence. The medium was changed to serum-free DMEM prior to treatment with microsporidia. After 48 h of microsporidia infection, cell resistance was measured by multifunctional electricity meter. One electrode was put into the medium of the upper chamber, while the other one was put into the medium of the lower chamber. The readings were recorded after the instrument stabilized and the resistance was calculated based on the area of the culture insert.

### Cell viability

Caco-2/MDCK cells were incubated on 12-well plates (1 × 10^5^ cells per well) at 37℃, and then the microsporidia were added for 48 h. Survival curves of the cells were assessed by cell counting kit 8 (Bioground, CAS 193149–74-5, China). Incubate the cells by mixing 1,000 µL DMEM and 100 µL of CCK-8 reagent for 30 min, and the plates were read at 450 nm.

### Wound healing assay

Caco-2 and MDCK cells were infected by microsporidia for 48 h, cell monolayers were scratched with a pipette tip, washed with phosphate-buffered saline (PBS) three times, followed by incubation with DMEM containing 10%FBS. Photographs of the wound and the healing processes were recorded by microscope (Zeiss, Oberkochen, Germany). The percentages of wound healing with time were assessed and calculated by ImageJ software (Bethesda, MD, USA). Experiments were performed in duplicate, with five measure-sites per scratch (55).

### Hematoxylin and eosin (H&E) staining

The small intestinal tissue of mice was fixed with 4% paraformaldehyde and embedded after dehydration of ethanol with different gradients. For paraffin-embedded tissue, sections (4 µm) were stained with H&E.

### Immunofluorescent assays

Control or microsporidia-infected MDCK/Caco-2 cells were fixed with 4% paraformaldehyde and permeabilized by 0.1% Triton X-100. Tight junctions were visualized by using anti-ZO-1 rabbit antibody (Cat # 21773–1-AP, Proteintech, USA; Diluted 1:1000) and fluorescent secondary antibody by immunofluorescent microscopy. For visualization of microsporidia spores and proliferation within cells, the fixed cell samples were incubated overnight at 48℃ with hybridization buffer containing 3 ng/µL of microsporidia-specific RNA probes (Cy3-GTTCTCCTGCCCGCTTCA and Cy3-ACTCTCACACTCACTTCAG for *E. intestinalis* and *E. hellem*, respectively; Sangon Biotech, China). Laser scanning confocal microscopy was performed with Olympus FV1200 laser scanning confocal microscope (Olympus, Tokyo, Japan).

### DSS treatment and fecal bleeding detection

The DSS-treated group of mice received DSS after microsporidia infection. Mice were treated with DSS (3.5%) in the drinking water for 4 days and libitum on water for 3 days. Afterward, the feces of all groups of mice were collected and assayed by fecal occult blood detection kit (Leagene, Cas 9011–18-1, China) for three consecutive days to assess bleeding. Body weights of all groups were also monitored.

### Gut microbiota analysis

Stool samples were collected from microsporidia, either *E. hellem* or *E. intestinalis*, infected mice, or control ones. OMEGA Soil DNA Kit (D5625-01) (Omega Bio-Tek, Norcross, GA, USA) extracts genomic DNA from samples and tests the purity and concentration of DNA. The following analysis was accomplished with the aid of Shanghai Applied Protein Technology Co., Ltd (APTBIO, China). According to the selection of sequencing region, the selected V3-V4 variable region was amplified by polymerase chain reaction and the amplification recovered products were detected and quantified with the Microplate reader (BioTek, FLx800) fluorescence quantitative system. The library was constructed using TruSeq Nano DNA LT Library Prep Kit from Illumina. The constructed library is inspected by Agilent Bioanalyzer 2100 and Promega QuantiFluor. After the library is qualified, it is sequenced. Raw sequencing data were in FASTQ format. Paired-end reads were then preprocessed using Cutadapt software to detect and cut off the adapter. Cutadapt filters and removes low-quality sequences such as adapter sequences, primers, poly-A tails, and other types of unwanted sequences from the high-throughput sequences. The Cutadapt-based microbiome analysis pipeline was then performed and accomplished in QIIME2. Both 16S rDNA amplicons and internal transcribed spacer were sequenced. To compare the dissimilarity of microbiota community compositions after microsporidia infection, the beta diversity was calculated with QIIME2.

### Statistics analysis

For statistical analysis and group comparison, one-way analysis of variance and Student’s *t* test were implemented to figure out the difference. *P* < 0.05 was deemed to be an indication of significant difference between the two groups.

## Data Availability

The full set of mouse gut microbiome data (for both bacteria and fungi metagenome raw sequence reads) has been deposited in the NCBI Sequence Read Archive (SRA) under accession numbers PRJNA1046261PRJNA1046261 and PRJNA1046648.
